# Supplementary material for: MetaRibo-Seq measures translation in microbiomes
Source: Nat Commun. 2020 Jun 29;11:3268. doi: 10.1038/s41467-020-17081-z (PMC7324362; doi:10.1038/s41467-020-17081-z)
Supplement: Supplementary file 10 — Supplementary Data 7 [file 41467_2020_17081_MOESM10_ESM.zip › File2/Confidence_VeryHigh_Taxonomy/88645_out.krona.html]

Javascript must be enabled to view this page.

members
magnitude
magnitudeUnassigned
count
unassigned
taxon
rank

88645\_out

5

2
superkingdom
5

5
phylum
1239

186801
class
5

186802
order
5

5
186803
family

genus
1164882
1

1

SRS019607\_contig\_number\_22489
979627
species

2

SRS017120\_contig\_number\_contig-100\_4217.102445SRS024318\_contig\_number\_contig-100\_8805.193398.193398
712976
species

1
genus
841


SRS049959\_contig\_number\_28210
1
360807
species

1843210
genus
1

1527
species
1

SRS021484\_contig\_number\_5185
